# ERα36 is associated with SLC7A5/LAT1 expression, cervical cancer progression, and BNCT response: a translational study

**DOI:** 10.3389/fonc.2026.1902977

**Published:** 2026-08-18

**Authors:** Lili Zhang, Rui Quan, Hefa Huang, Wei Wang, Lu Zhang, Long Gu, Zhongying Dai

**Affiliations:** 1 Reproductive Medicine Center, The First Hospital of Lanzhou University, Lanzhou, China; 2 School of Nuclear Science and Technology, Lanzhou University, Lanzhou, China; 3 Frontiers Science Center for Rare Isotopes, Lanzhou University, Lanzhou, China

**Keywords:** boron neutron capture therapy (BNCT), cervical squamous cell carcinoma, ERα36, SLC7A5/LAT1, tumor microenvironment

## Abstract

**Objective:**

To elucidate the role and molecular mechanism of the ERα36–SLC7A5/LAT1 signaling axis in the malignant progression of cervical cancer and its sensitivity to boron neutron capture therapy (BNCT).

**Methods:**

Transcriptomic data from TCGA (n=296) and GTEx (n=10) were integrated to analyze SLC7A5 expression and its clinicopathological associations. Multiplex immunofluorescence staining was performed on a tissue microarray containing 99 cervical cancer specimens for spatial phenotyping. SiHa cell lines with stable ERα36 knockdown or overexpression were established using lentiviral transduction. CCK-8, flow cytometry, wound healing, Transwell, and colony formation assays were used to assess the effects of ERα36 on malignant phenotypes. Co-immunoprecipitation, Western blot, and qRT-PCR were employed to explore the interaction between ERα36 and LAT1 as well as downstream signaling changes. The impact of ERα36 on BNCT sensitivity was evaluated in both cellular and subcutaneous xenograft nude mouse models.

**Results:**

SLC7A5 was significantly upregulated in cervical cancer tissues and correlated with M stage and poor prognosis. ERα36 and SLC7A5 exhibited robust spatial co-expression in the tumor microenvironment, and the double-positive phenotype was associated with shorter overall survival (P<0.05). ERα36 interacted with LAT1 and was associated with elevated CD98hc and EGFR expression. Knockdown of ERα36 suppressed proliferation, migration, and colony formation while promoting apoptosis; overexpression produced opposite effects. In BNCT simulation experiments, ERα36 knockdown markedly attenuated BNCT-induced cytotoxicity, as evidenced by increased colony formation, reduced apoptosis, and altered S-phase distribution. Both cellular and animal models consistently showed downregulation of ERα36 and LAT1 protein expression after BNCT.

**Conclusion:**

The ERα36–SLC7A5/LAT1 axis associated with malignant phenotypes and BNCT sensitivity in cervical cancer. The ERα36+/SLC7A5+ spatial double-positive phenotype may represent a prognostic biomarker, and ERα36 may serve as a predictive marker for BNCT response.

## 1 Introduction

Cervical cancer ranks among the most burdensome malignancies globally for women (1). While the widespread implementation of HPV vaccination and cytological screening has markedly reduced its incidence, the risk of recurrence and distant metastasis post-initial treatment remains persistently high in patients with advanced disease. Notably, for those who develop secondary resistance following radical chemoradiotherapy or cannot complete the prescribed regimen due to treatment-related toxicity, there is an urgent need to identify novel stratification indices that are both biologically informative and clinically actionable, and to develop tailored intervention strategies accordingly (2). However, single molecular biomarkers often fail to systematically capture the intrinsic clonal heterogeneity, microenvironmental status, and dynamic therapeutic response profiles of tumors (3).

Within the overarching framework of tumor metabolic reprogramming, amino acid transport systems are increasingly recognized as highly promising therapeutic targets. The L-type amino acid transporter 1 (LAT1, encoded by SLC7A5) typically forms a heterodimeric complex with the heavy chain CD98hc (SLC3A2), mediating the transmembrane transport of neutral essential amino acids such as leucine (4). Classic studies have demonstrated that this complex precisely regulates nutrient-sensing pathways (e.g., mTORC1) via a “glutamine efflux-leucine influx” exchange mechanism, thereby supporting tumor cell protein synthesis, proliferation, and survival advantages (5, 6). A growing body of preclinical and clinical evidence indicates that high LAT1 expression in various solid tumors correlates with aggressive phenotypes and poor prognosis (4, 7, 8). In cervical cancer, LAT1 expression exhibits striking spatiotemporal heterogeneity: it is relatively downregulated in certain high-grade squamous intraepithelial lesions but significantly upregulated in invasive carcinoma tissues, especially at the tumor invasion front, and is associated with epithelial–mesenchymal transition acquisition and enhanced stromal invasion capacity (9). This characteristic suggests that the functional significance of LAT1 extends beyond tumor initiation, reflecting adaptive metabolic reprogramming of the tumor under microenvironmental selection pressures such as nutrient deprivation and hypoxia.

Beyond metabolic reprogramming, hormone–receptor signaling pathways play a critical and synergistic role in the progression of HPV-associated cervical cancer. Preclinical evidence from genetically engineered mouse models demonstrates that estrogen and its nuclear receptor ERα cooperatively drive malignant transformation of the cervical epithelium in the context of persistent HPV oncogene expression (10–12). Yet, clinical specimens exhibit marked heterogeneity in both expression levels and transcriptional activity of canonical nuclear ERα (ERα66), implying the existence of alternative estrogen signaling paradigms—such as receptor isoform switching or preferential engagement of non-genomic signaling cascades (10). ERα36, a membrane-anchored splice variant of the ESR1 gene that lacks the AF-1 and AF-2 transactivation domains, mediates rapid ligand-dependent activation of cytoplasmic kinase signaling networks, including the MAPK/ERK and PI3K/AKT pathways, thereby promoting cell migration, invasion, proliferation, and resistance to anticancer therapies (13–15). Notably, ERα36 physically interacts with EGFR and HER2 to form hybrid receptor complexes that sustain bidirectional cross-activation, amplifying downstream oncogenic signaling and conferring resistance to endocrine and targeted agents (16–18).

Despite their well-documented individual roles in cervical carcinogenesis, a direct molecular link between ERα36 and LAT1 remains uncharacterized. Given their shared plasma membrane localization, overlapping functional outputs such as enhanced proliferation, motility, and cell survival, and convergent regulation of key oncogenic signaling nodes such as mTORC1 and EGFR, we hypothesize that ERα36 may functionally regulate LAT1 expression, protein stability, or transport activity. Elucidating this axis would uncover a previously unrecognized node of crosstalk between steroid hormone signaling and amino acid metabolism, a nexus that is potentially central to tumor adaptation and therapeutic vulnerability.

Importantly, LAT1 serves not only as a metabolic regulator but also as the principal functional gateway for boron delivery in boron neutron capture therapy (BNCT). BNCT is a binary, biologically targeted radiotherapy: the ¹^0^B-enriched amino acid analog boronophenylalanine (BPA) relies almost exclusively on LAT1-mediated transport for selective intratumoral accumulation; subsequent thermal or epithermal neutron irradiation triggers high-linear-energy-transfer (LET) α-particle and ^7^Li recoil nucleus emissions, resulting in spatially confined, highly cytotoxic DNA damage (19, 20). Robust experimental and clinical data confirm that LAT1 expression level and membrane trafficking efficiency are rate-limiting determinants of BPA uptake, intracellular boron concentration, and ultimately, BNCT-induced clonogenic cell death (21) ^(^22^),^. While preliminary *in vitro* and xenograft studies report measurable BPA accumulation and radiosensitization in cervical cancer models (23), the upstream molecular determinants governing intertumoral heterogeneity in BNCT response remain undefined.

Collectively, this study establishes and mechanistically interrogates the ERα36–SLC7A5/LAT1 signaling axis in cervical cancer. We systematically address three interrelated questions (1): What is the clinicopathological significance and prognostic value of SLC7A5/LAT1 expression across cervical cancer stages? (2) Do ERα36 and LAT1 exhibit spatially coordinated co-expression and physical interaction within the tumor microenvironment—and if so, what is the biochemical nature and functional consequence of this association? (3) Does ERα36 modulate malignant phenotypes and BNCT sensitivity in a LAT1-dependent manner? By integrating spatial biology, molecular interaction assays, and functional BNCT modeling, this work reveals a novel hormone–metabolism regulatory axis that governs both tumor progression and radiation therapeutic response, thereby providing a mechanistic foundation for biomarker-driven patient stratification and rational combination strategies involving endocrine modulation and BNCT.

## 2 Materials and methods

### 2.1 Data sources and bioinformatics analysis

In this study, transcriptome data, clinical pathological information, and survival data of cervical squamous cell carcinoma (CESC) were downloaded from The Cancer Genome Atlas (TCGA) database. After screening, a total of 296 patient samples with complete information were included. Normal cervical tissue control data were obtained from the Genotype-Tissue Expression (GTEx) database, including 10 samples. TCGA RNA-Seq data were normalized using the FPKM (Fragments Per Kilobase of transcript per Million mapped reads) method, and GTEx data were processed via a unified pipeline. Batch effects between the two datasets were corrected using the ComBat algorithm implemented in the R package “sva” prior to differential expression analysis. All data analyses were performed in R language (version 4.0.3). The Wilcoxon rank sum test was used to compare the expression of SLC7A5 between tumor and normal tissues. The association between its expression level and clinical pathological parameters was analyzed using the Kruskal-Wallis test or Wilcoxon test. The survminer package’s surv_cutpoint function was used to determine the optimal cutoff value of SLC7A5 expression, and patients were divided into high and low expression groups. Survival curves were plotted using the Kaplan-Meier method, and differences between groups were compared using the Log-rank test.

### 2.2 Clinical samples and tissue microarray

The tissue microarray (TMA, catalog number: HUteS111Su01) used in this study was purchased from Shanghai Aovier Biotechnology Co., Ltd. The TMA contains 99 cervical cancer tissue samples and 12 paired adjacent normal tissues. All original samples were collected with informed consent from the participants and approved by the Ethics Committee of the supplier (Approval No. 2005DKA21300). The samples were anonymized prior to provision, rendering the donors unidentifiable to the researchers. This study was conducted in strict accordance with the fundamental principles of the Declaration of Helsinki. Clinicopathological data for the 99 cervical cancer patients, including age, histological type, histological grade, T stage, N stage, M stage, clinical stage (AJCC 7th edition), HPV status, follow-up status, overall survival, disease-free survival, and recurrence/progression events, were extracted from the pathology reports and clinical follow-up records provided by the supplier. The detailed clinicopathological characteristics and follow-up information are summarized in **Supplementary Table 1**. For cases with missing data, the proportion of missingness is noted in the table footnote. Data were collected with a follow-up cutoff date of March 2017. For multiplex immunofluorescence staining, TMA sections were dewaxed, subjected to antigen retrieval using AR9 buffer (high pH, pH 9.0 Tris-EDTA) in a microwave, and treated with a peroxidase blocker. The sections were then incubated with primary antibodies at room temperature for 1 hour, followed by secondary antibody (SM802, ready-to-use, DAKO) and Opal fluorescent dyes (Opal 7-color kit, PerkinElmer). Antibody stripping was performed by microwave treatment to enable multiple labeling. Nuclei were counterstained with DAPI and the sections were mounted. Detailed antibody information—including manufacturer, catalog number, dilution, Opal dye, and antigen retrieval method—is provided in **Supplementary Table 2**. All antibodies were validated on cervical cancer tissue microarrays with appropriate positive and negative controls. Quantitative image analysis was performed using StrataQuest software (version 7.1.129, TissueGnostics GmbH, Vienna, Austria). Valid nuclei were identified based on DAPI staining, with debris excluded by fluorescence intensity versus area gating. Protein signals were located by setting a distance radius around valid nuclei, and positive cell populations were gated based on fluorescence intensity and area. Double-positive cells were identified by dual-channel fluorescence intensity scatter plots (upper-right quadrant for A+B+). Tumor epithelium and stroma were segmented based on CK staining, with boundary cells assigned using a nuclear intensity threshold of 127. All thresholds were established using isotype and FMO controls, applied uniformly across all samples, and the analysis was performed blinded to clinical outcomes. All statistical analyses of TMA data were performed at the patient level using summarized cell counts per patient (proportions or densities) rather than treating individual cells as independent samples, as detailed in Section 2.15. In accordance with journal guidelines, we will make the raw data available to the editorial team for independent analysis or reproducibility purposes.

### 2.3 Multiple immunofluorescence staining

After dewaxing and antigen retrieval of the tissue microarray sections, they were treated with a peroxidase blocker. The sections were then incubated with the following primary antibodies at room temperature for 1 hour: anti-ERα (catalog number AF6058, 1:200), anti-ERα36 (catalog number DZ096-874, 1:200), anti-SLC7A5 (catalog number DF8065, 1:200), and anti-Ki67 (catalog number ZM0166, 1:200). After washing, they were incubated with a secondary antibody (SM802, ready-to-use, DAKO) and Opal fluorescent dyes (Opal 7-color kit, PerkinElmer). Antibody stripping was performed by microwave treatment to enable multiple labeling. Finally, the nuclei were counterstained with DAPI and the sections were mounted.

### 2.4 Image acquisition and analysis

Images were acquired using the Zeiss AXIOSCAN 7 whole-slide scanner at a resolution of 0.22 μm/pixel. Quantitative analysis was performed using StrataQuest analysis software (version 7.1.129, TissueGnostics): nuclei were identified and segmented based on DAPI signals, fluorescence intensity thresholds for each channel were set, and single-positive and multi-positive cells were automatically identified. The number, fluorescence intensity, and spatial co-localization parameters of these cells were calculated.

### 2.5 Cell culture

Human cervical cancer cell lines SiHa, HeLa, radiotherapy-resistant SiHa cells, and normal cervical epithelial cells H8 were all purchased from the American Type Culture Collection (ATCC). All cells were cultured in DMEM medium supplemented with 10% fetal bovine serum at 37°C in a 5% CO_2_ incubator.

### 2.6 Construction of stable cell lines

Stable SiHa cell lines with ERα36 knockdown and overexpression were constructed using lentiviral transfection technology. shRNA sequences (including sh1, sh2, sh3, and a non-targeting control) and overexpression sequences targeting ERα36 were designed and synthesized, and then cloned into lentiviral vectors. The specific shRNA sequences are provided in **Supplementary Table 3**. After virus packaging and infection, stable cell lines were selected using puromycin. The knockdown and overexpression efficiencies were verified by Western Blot and qRT-PCR, and the sh2 cell line with the highest knockdown efficiency was selected for subsequent experiments.

### 2.7 Western blot analysis

Total proteins were extracted from cells using RIPA lysis buffer, and the concentration was determined by the BCA method. Equal amounts of protein were separated by 10% SDS-PAGE and transferred to PVDF membranes. After blocking, the membranes were incubated with primary antibodies at 4°C overnight. The primary antibodies used included anti-ERα36, anti-LAT1, anti-CD98hc, anti-EGFR, and the internal reference anti-GAPDH. The next day, the membranes were incubated with HRP-conjugated secondary antibodies, and detected by ECL chemiluminescence.

### 2.8 Co-immunoprecipitation

Cell lysates were incubated with anti-ERα36 antibody or isotype control IgG at 4°C overnight, and then Protein A/G agarose beads were added for further incubation for 4 hours. The precipitated complexes were washed and the interacting LAT1 protein was detected by Western Blot.

### 2.9 RNA extraction and real-time quantitative PCR

Total RNA was extracted from cells using TRIzol reagent and reverse transcribed into cDNA. Real-time quantitative PCR amplification was performed using SYBR Green Master Mix. GAPDH was used as an internal reference, and the relative expression level was calculated using the 2^(-ΔΔCt) method. All reactions were performed in triplicate.

### 2.10 Cell proliferation and colony formation assay

Cell proliferation was detected using the CCK-8 kit, and the absorbance was measured at 450 nm. For the colony formation assay, 500 cells per well were seeded in 6-well plates and cultured for 10-14 days. The colonies were fixed with methanol, stained with crystal violet, and the number of colonies containing more than 50 cells was counted.

### 2.11 Cell migration assay

After cells reached confluence, a scratch was made with a 200 μL pipette tip. The width of the scratch was observed and measured at 0, 24, and 48 hours. Transwell migration assay: 5×10_4_ cells were seeded in the upper chamber of a Transwell insert in serum-free medium, and the lower chamber was filled with medium containing 10% FBS. After 24 hours of culture, the cells were fixed, stained, and the number of migrated cells was counted.

### 2.12 Cell apoptosis detection

Cells from each group (approximately 1×10_6_) were collected, washed with PBS, and resuspended in binding buffer. Annexin V-FITC and PI staining solution were added, and the samples were incubated in the dark at room temperature for 15 minutes. Apoptosis was detected by flow cytometry (BD FACSCanto II), and the proportion of early and late apoptotic cells was analyzed using FlowJo software.

### 2.13 Cell cycle analysis

After collecting the cells, they were fixed with 70% ethanol at 4°C overnight. The cells were stained with PI staining solution in the dark for 30 minutes. DNA content was detected by flow cytometry, and the cell cycle distribution ratio was analyzed using ModFit LT software.

### 2.14 Boron neutron capture therapy experiment

The accelerator-based boron neutron capture therapy (AB-BNCT) system independently developed by Lanzhou University was used for irradiation experiments. The proton beam energy was 2.6 MeV, and the beam current was 6 mA. The system measured a medium-energy neutron flux of 6.96×10_8_ n/(cm²·s) at 2 cm from the beam exit. Cells were seeded in 6-well plates and incubated with 26 ppm BPA for 6 hours. After incubation, the BPA-containing medium was removed, and cells were rinsed once with physiological saline. Subsequently, 500 μL of concentrated nitric acid was added to each well, followed by digestion at 60°C for 0.5 h. After digestion, 2.5 mL of distilled water was added to each well and mixed thoroughly. Boron content in each sample was quantified using inductively coupled plasma atomic emission spectrometry (ICP-AES; model PQ 9000, Analytik Jena, Germany). Boron concentration was normalized to total protein content (measured by BCA assay) to account for differences in cell number. Cells were seeded and allowed to adhere overnight, then incubated with 26 ppm BPA)for 6 hours prior to irradiation. The following treatment groups were established: untreated control (N0); neutron irradiation alone for 10 min (N10) or 20 min (N20); BPA alone at 26 ppm (BPA); and BPA combined with neutron irradiation for 10 min (BPA+N10) or 20 min (BPA+N20). After irradiation, cells were replenished with fresh medium and cultured for 48 hours, after which colony formation assays, apoptosis detection, and cell cycle analysis were performed as described above. All experiments were independently repeated three times.

### 2.15 Statistical analysis

Bioinformatics analyses were performed using R software (version 4.0.3). For TCGA and GTEx data, SLC7A5 expression differences between tumor and normal tissues were assessed using the Wilcoxon rank-sum test, and associations with clinicopathological parameters were evaluated using the Kruskal-Wallis or Wilcoxon test. For multiple comparisons across clinicopathological variables, Benjamini-Hochberg false discovery rate (FDR) correction was applied. Survival curves were generated using the Kaplan-Meier method with Log-rank tests. For tissue microarray (TMA) data, all statistical analyses were performed at the patient level using summarized cell counts per patient (proportions or densities), not at the individual-cell level. Group comparisons between tumor and adjacent normal tissues were performed using the Wilcoxon signed-rank test for paired samples. Correlation between marker expression levels was assessed using Pearson correlation analysis. Survival analyses for TMA data were conducted using the Kaplan-Meier method with Log-rank tests, and prognostic factors were evaluated using univariate and multivariate Cox proportional hazards regression models, with hazard ratios (HR) and 95% confidence intervals (CI) reported. Sample size for animal experiments (n = 5 per group) was determined by power analysis using G*Power software (version 3.1), assuming an effect size of 1.5-fold difference in tumor volume between treatment and control groups, with α = 0.05 and statistical power (1-β) = 0.80. For cell experiments, data are presented as mean ± standard deviation (SD) and analyzed using GraphPad Prism 9.0 software. Normality was assessed using the Shapiro-Wilk test, and equality of variances was assessed using Levene’s test. Comparisons between two groups were performed using Student’s t-test (or Welch’s t-test when variances were unequal), and among multiple groups using one-way ANOVA followed by Tukey’s post-hoc test. For BNCT experiments with two variables (genotype and treatment), two-way ANOVA with Tukey’s post-hoc test was applied, with outcomes normalized to the corresponding untreated genotype control. Cell experiments were performed with at least three independent biological replicates (n=3). For the clinical TMA cohort, sample size (n=99) was determined by the availability of the commercial tissue microarray. A P value < 0.05 was considered statistically significant. All statistical tests were two-sided.

## 3 Results

### 3.1 SLC7A5/LAT1 is upregulated in cervical cancer and correlates with metastasis and poor prognosis

**Figure 1:**
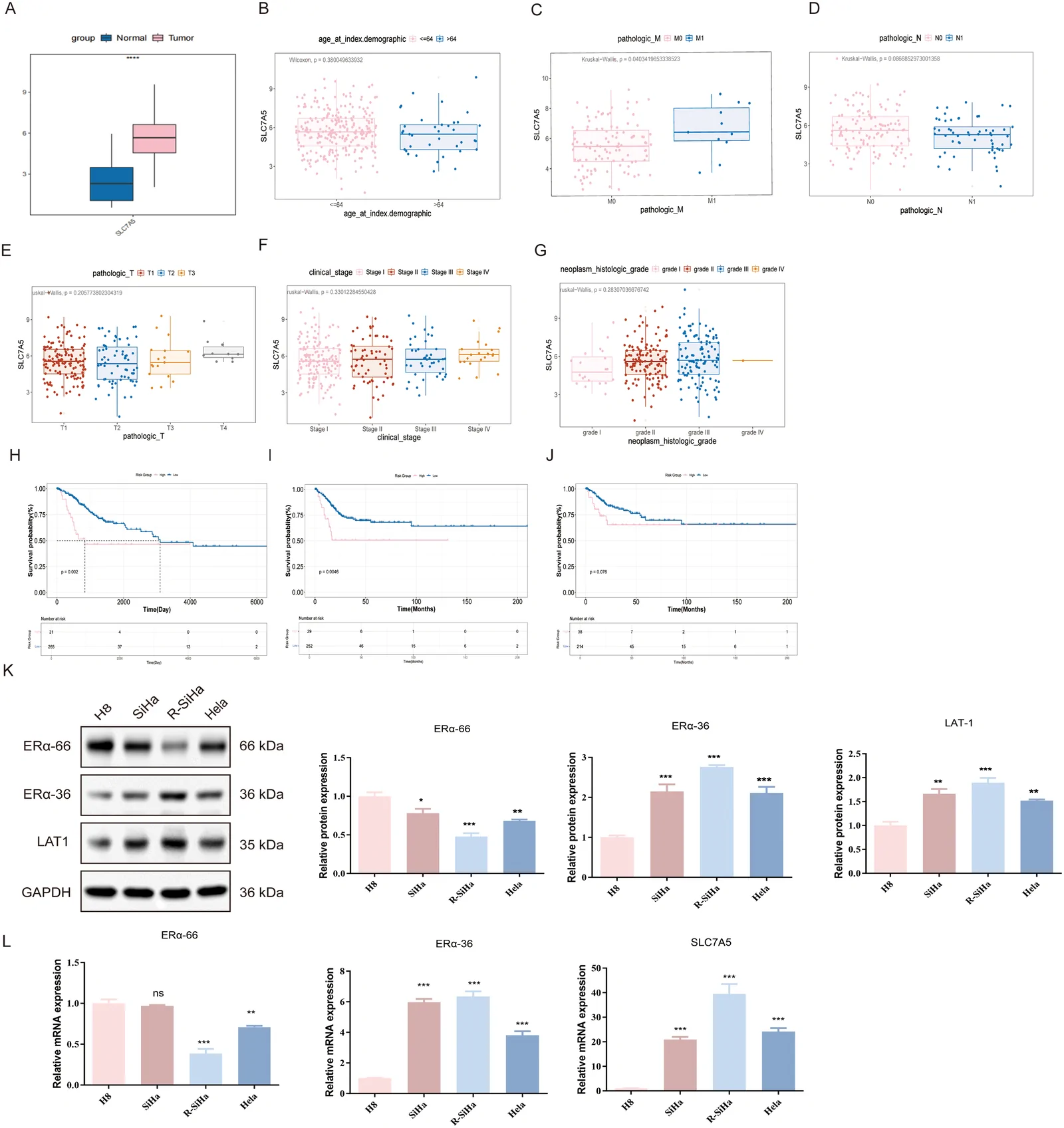
High expression of SLC7A5/LAT1 in cervical cancer is associated with poor prognosis. **(A)**. Comparison of SLC7A5 expression in cervical squamous cell carcinoma tissues from the TCGA database (n = 296) and normal cervical tissues from the GTEx database (n = 10). Data are presented as median and interquartile range, and statistical analysis was performed using the Wilcoxon rank sum test. **(B–G)** Association of SLC7A5 expression with clinicopathological parameters, including age **(B)**, pathologic M stage **(C)**, pathologic N stage **(D)**, pathologic T stage **(E)**, clinical stage **(F)**, and neoplasm histologic grade **(G)**. Statistical analysis was performed using Kruskal-Wallis or Wilcoxon tests as appropriate. **(H–J)** Kaplan–Meier survival curves based on SLC7A5 expression levels. The “surv_cutpoint” function was used to determine the optimal cut-off value, and patients were divided into high and low expression groups to analyze their relationship with overall survival [OS, **(H)**], progression-free survival [PFS, **(I)**], and disease-free survival [DFS, **(J)**]. Log rank test was used for comparison between groups. **(K)** Western blot detection of LAT1 protein expression levels in normal cervical epithelial cells H8 and cervical cancer cell lines (SIHA, radiotherapy-resistant SIHA, HeLa). GAPDH was used as the loading control. **(L)** qRT-PCR detection of LAT1 mRNA expression levels in the above cell lines. Data are the mean ± standard deviation of three independent experiments; *P < 0.05, **P < 0.01, ***P < 0.001.

To characterize the expression profile and clinical implications of SLC7A5/LAT1 in cervical cancer, transcriptomic data from 296 cervical squamous cell carcinoma (CESC) samples in The Cancer Genome Atlas (TCGA) and 10 normal cervical tissue samples from the Genotype-Tissue Expression (GTEx) project were integrated for analysis. Results demonstrated that SLC7A5 expression was significantly higher in cervical cancer tissues than in normal cervical tissues (P < 0.05, **Figure 1A**). Clinicopathological correlation analysis revealed a significant positive association between high SLC7A5 expression and distant tumor metastasis (M stage) (P < 0.05, **Figure 1C**), whereas no significant correlations were observed with patient age, clinical stage, pathological grade, T stage, or N stage (**Figures 1B, D–G**).

To further assess the prognostic value of SLC7A5, the optimal cutoff value for its expression was determined using the maximally selected rank statistic, stratifying the 296 patients into high- and low-expression groups. Kaplan-Meier survival analysis showed that the high SLC7A5 expression group had significantly shorter overall survival (OS, **Figure 1H**) and progression-free survival (PFS, **Figure 1I**) compared to the low-expression group (both P < 0.05), indicating that high SLC7A5 expression is strongly associated with poor prognosis in cervical cancer patients. However, no significant correlation was found between SLC7A5 expression and disease-free survival (DFS) (**Figure 1J**).

At the cellular level, Western blot and qRT-PCR assays confirmed that both LAT1 protein (encoded by SLC7A5) and its mRNA were significantly upregulated in cervical cancer cell lines (SiHa, HeLa, and radiotherapy-resistant SiHa) relative to normal cervical epithelial cells (H8) (**Figures 1K, L**). Notably, the upregulation of LAT1 was most pronounced in radiotherapy-resistant SiHa cells, suggesting its potential involvement in the mechanism of radiotherapy resistance in cervical cancer.

Additionally, the expression patterns of two estrogen receptor α (ERα) isoforms were examined. qRT-PCR results showed that ERα66 expression was downregulated, while ERα36 expression was significantly upregulated in cervical cancer cells compared to normal cervical cells (**Figures 1K, L**), consistent with previous studies. This finding implies that the two isoforms may exert distinct biological functions in cervical cancer.

### 3.2 ERα36 and SLC7A5 are co-expressed in the cervical cancer microenvironment and correlate with poor patient prognosis

**Figure 2:**
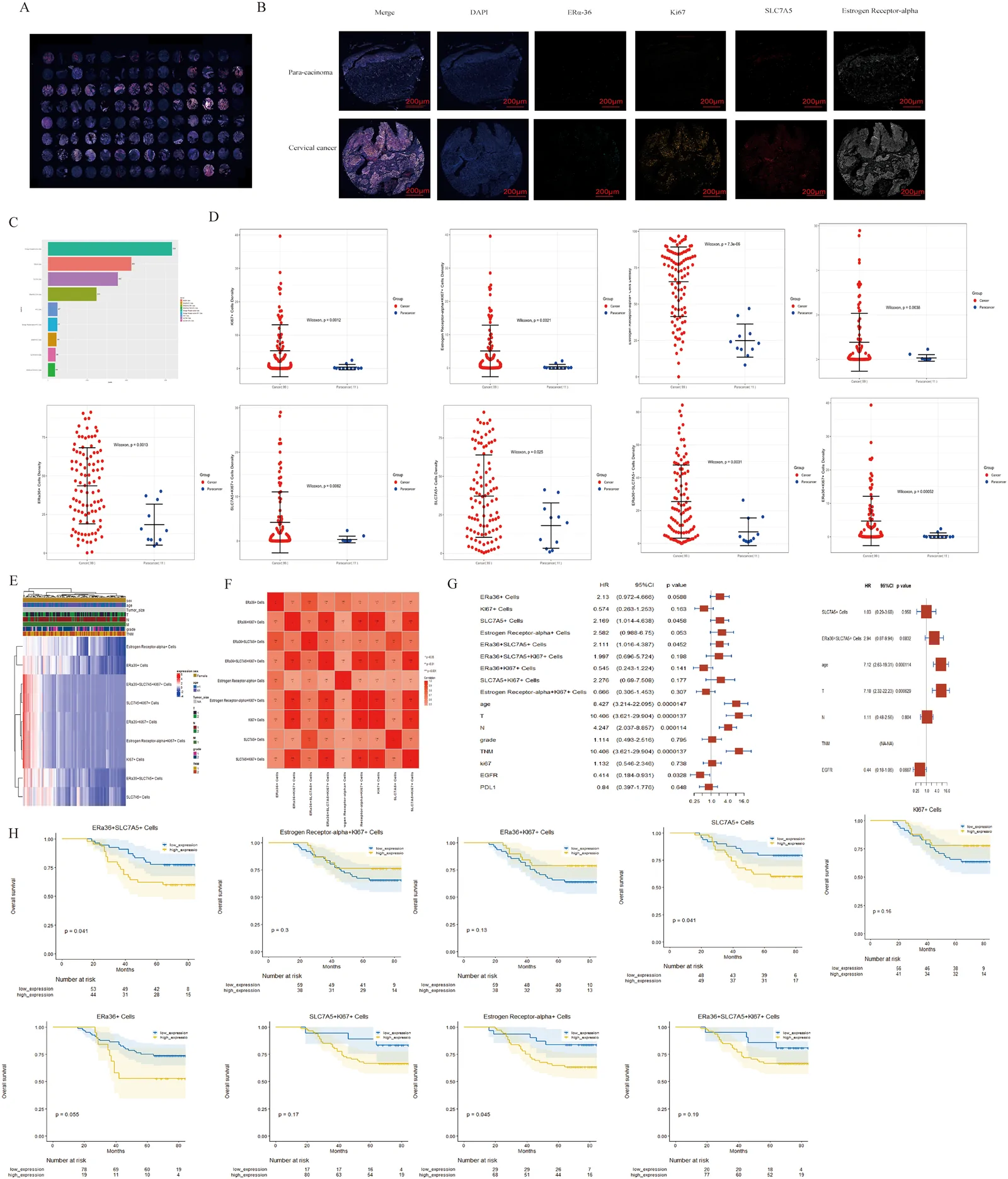
Characterization of the cervical cancer microenvironment based on multiplex immunofluorescence and co-expression and prognostic analysis of ERα36 and SLC7A5. **(A)** Representative images of multiplex immunofluorescence staining of cervical cancer tissue microarrays. Fluorescence signals of DAPI (blue, nuclei), ERα (white), ERα36 (cyan), SLC7A5 (red), and Ki67 (orange) are shown respectively. **(B)**. Comparison of representative images of multiplex immunofluorescence staining of cervical cancer tissues (left) and paired adjacent normal tissues (right) (magnification: 200×). **(C)** Bar chart of the distribution of different cell phenotypes in cervical cancer tissue samples. The length of the bars represents the count of positive cells for each phenotype, and the colors distinguish different cell phenotypes. **(D)** Comparison of the expression levels of each marker in tumor tissues and paired adjacent normal tissues. P < 0.05, P < 0.01, P < 0.001; ns indicates no statistical difference. **(E)** Cluster heatmap integrating the expression of multiple markers with clinical pathological features. Rows represent samples, and columns represent different cell phenotypes and clinical parameters. Red indicates high expression, and blue indicates low expression. Different types of clinical parameters are labeled above. **(F)** Pearson correlation heatmap of the expression levels of different cell phenotypes. Red indicates positive correlation, blue indicates negative correlation, and * indicates P < 0.05. **(G)** Forest plot of Cox regression analysis. The left side shows the results of univariate analysis of the ERα36+SLC7A5 double-positive phenotype; the right side shows the results of multivariate analysis including age, T stage, N stage, and the ERα36+SLC7A5 double-positive phenotype. The hazard ratio (HR) and 95% confidence interval (95% CI) of each factor are presented. **(H)** Kaplan-Meier survival curves based on the expression levels of key cell phenotypes.

To characterize the spatial expression patterns of ERα36 and SLC7A5 in cervical cancer tissues, multiplex immunofluorescence staining was performed on a tissue microarray comprising 99 tumor specimens and 12 paired adjacent normal tissues (**Figures 2A, B**). Quantitative analysis revealed 2,474 ERα36+/SLC7A5+ double-positive cells, representing 69.5% of all SLC7A5-positive cells—indicating robust co-expression of the two proteins in cervical cancer (**Figure 2C**). Spatial phenotyping further demonstrated heterogeneous distribution of distinct cellular subpopulations across tumor regions (**Figure 2C**).

Compared with matched adjacent normal tissues, cervical tumors exhibited significantly higher proportions of Ki67+, ERα+, ERα36+, and SLC7A5+ cells, as well as composite phenotypes including ERα36+/SLC7A5+, ERα36+/Ki67+, and SLC7A5+/Ki67+ (**Figure 2D**; all P < 0.05). Unsupervised hierarchical clustering integrating multi-marker expression profiles with clinicopathological variables revealed that specific cellular phenotypes clustered significantly with advanced clinical stage and higher histological grade (**Figure 2E**). Moreover, SLC7A5 and ERα36 expression levels showed a strong positive correlation within the tumor microenvironment (Pearson r = 0.575, P < 0.001; **Figure 2F**), suggesting potential functional interdependence or shared upstream regulation.

To evaluate the prognostic value of immunophenotypes and clinicopathological parameters for overall survival, univariate and multivariate Cox regression analyses were performed, with results presented as forest plots (**Figure 2G**). the complete regression results for all variables are provided in **Supplementary Table 4**. Univariate analysis revealed that SLC7A5 single-positive cells (HR = 2.169, 95% CI: 1.014–4.638, P = 0.0458) and the ERα36+/SLC7A5+ double-positive phenotype (HR = 2.111, 95% CI: 1.016–4.387, P = 0.0452) were significantly associated with worse overall survival. Among clinical parameters, age, T stage, N stage, TNM stage, and low EGFR expression were also significantly correlated with adverse survival outcomes. In the multivariate model adjusted for the above significant variables, only age and T stage remained independent adverse prognostic factors (both P < 0.001). Notably, the statistical significance of the ERα36+/SLC7A5+ double-positive phenotype was lost, although it retained a borderline trend toward increased risk (HR = 2.94, 95% CI: 0.87–9.94, P = 0.0832). The SLC7A5 single-positive phenotype showed no predictive value (HR = 1.03, 95% CI: 0.29–3.68, P = 0.958). These results suggest that neither cellular phenotype independently predicts patient survival, likely due to collinearity with the clinical parameters adjusted in the model, such as age and tumor T stage. Kaplan–Meier analysis demonstrated that patients with high SLC7A5 expression, ERα36+/SLC7A5+ co-expression, or high ERα expression exhibited significantly shorter overall survival compared with their low-expression counterparts (Log-rank test, all P < 0.05; **Figure 2H**). Collectively, these findings indicate that ERα36 and SLC7A5 are frequently co-expressed in the cervical cancer microenvironment and that their co-expression is associated with aggressive disease and poor clinical outcome, thereby supporting their potential utility as complementary prognostic biomarkers.

### 3.3 ERα36 drives malignant phenotypes in cervical cancer cells by enhancing proliferation and migration, suppressing apoptosis, and facilitating clonogenic survival

**Figure 3:**
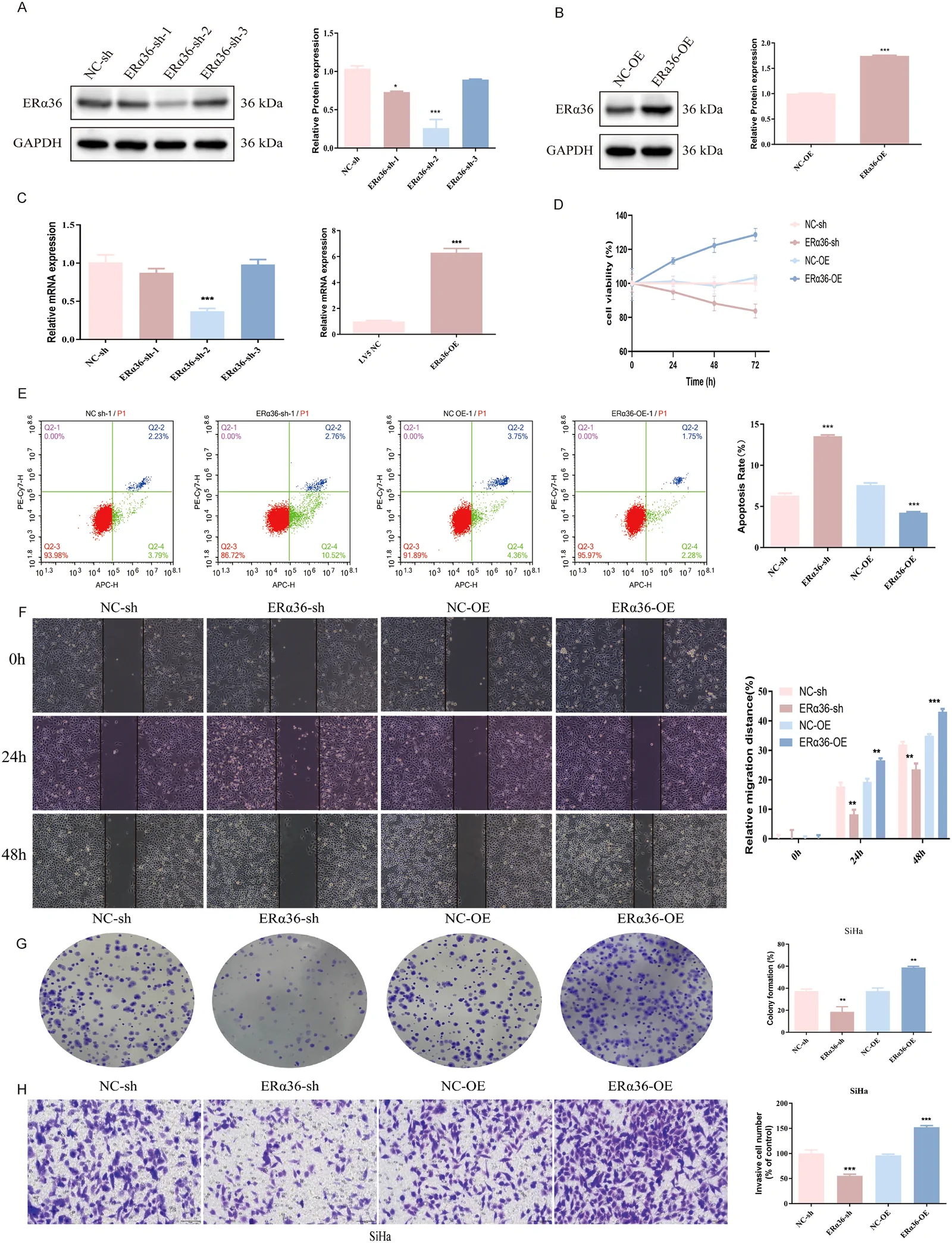
Functional study of ERα36 in regulating malignant phenotypes in cervical cancer cells. **(A–C)** Construction and validation of stable ERα36 knockdown and overexpression cell lines. **(A)** Western blot analysis of ERα36 protein expression levels after knockdown with different shRNA sequences (sh1, sh2, sh3); **(B)** Western blot analysis of ERα36 protein expression levels after overexpression (OE); **(C)** qRT-PCR analysis of ERα36 mRNA expression levels in knockdown (sh2) and overexpression cell lines. Data are presented as mean ± standard deviation (n = 3), *P < 0.05, **P < 0.01, ***P < 0.001. **(D)** CCK-8 assay to analyze the effect of ERα36 knockdown or overexpression on the proliferation ability of SiHa cells. **(E)** Annexin V-FITC/PI double staining flow cytometry to detect the effect of ERα36 expression alteration on cell apoptosis (n = 3, ***P < 0.001). **(F)** Scratch assay to evaluate the effect of ERα36 on cell migration ability, with observations and calculations of scratch healing rates at 0, 24, and 48 hours (scale bar: 200 μm, n = 3, **P < 0.01, ***P < 0.001). **(G)** Colony formation assay to analyze the effect of ERα36 knockdown or overexpression on cell colony formation ability (crystal violet staining, n = 3, **P < 0.01). **(H)** Transwell migration assay to further verify the regulatory effect of ERα36 on cell migration (crystal violet staining, scale bar: 100 μm, n = 3, ***P < 0.001).

To investigate the functional role of ERα36 in cervical cancer, we established SiHa cell lines with stable ERα36 knockdown (sh2) or ectopic overexpression. Validation by Western blot and qRT-PCR confirmed efficient and specific modulation of ERα36 expression: sh2 cells exhibited marked reduction in both ERα36 protein and mRNA levels relative to control cells (**Figures 3A, C**), whereas ERα36-overexpressing cells showed robust upregulation (**Figures 3B, C**). Given its superior knockdown efficiency, the sh2 clone was selected for all subsequent functional assays.

A comprehensive panel of functional assays revealed that ERα36 critically regulates multiple hallmarks of malignancy. CCK-8-based proliferation assays demonstrated that ERα36 knockdown significantly suppressed cell growth, while its overexpression accelerated proliferation (**Figure 3D**). Annexin V-FITC/PI staining followed by flow cytometry revealed that ERα36 depletion increased both early and late apoptotic populations, whereas ERα36 overexpression conferred resistance to apoptosis (**Figure 3E**). Consistent results were obtained across complementary migration assays: both wound healing (scratch) and Transwell assays showed that ERα36 knockdown markedly impaired migratory capacity, whereas overexpression enhanced directional motility (**Figures 3F, H**). Furthermore, colony formation assays indicated that ERα36 deficiency reduced the number and size of colonies, while its overexpression augmented clonogenic potential (**Figure 3G**). Collectively, these data establish that ERα36 functions as a potent oncogenic driver in cervical cancer cells—promoting proliferation and migration, inhibiting apoptosis, and enhancing long-term clonogenic survival.

### 3.4 ERα36 physically interacts with LAT1 and correlates with CD98hc and EGFR expression

**Figure 4:**
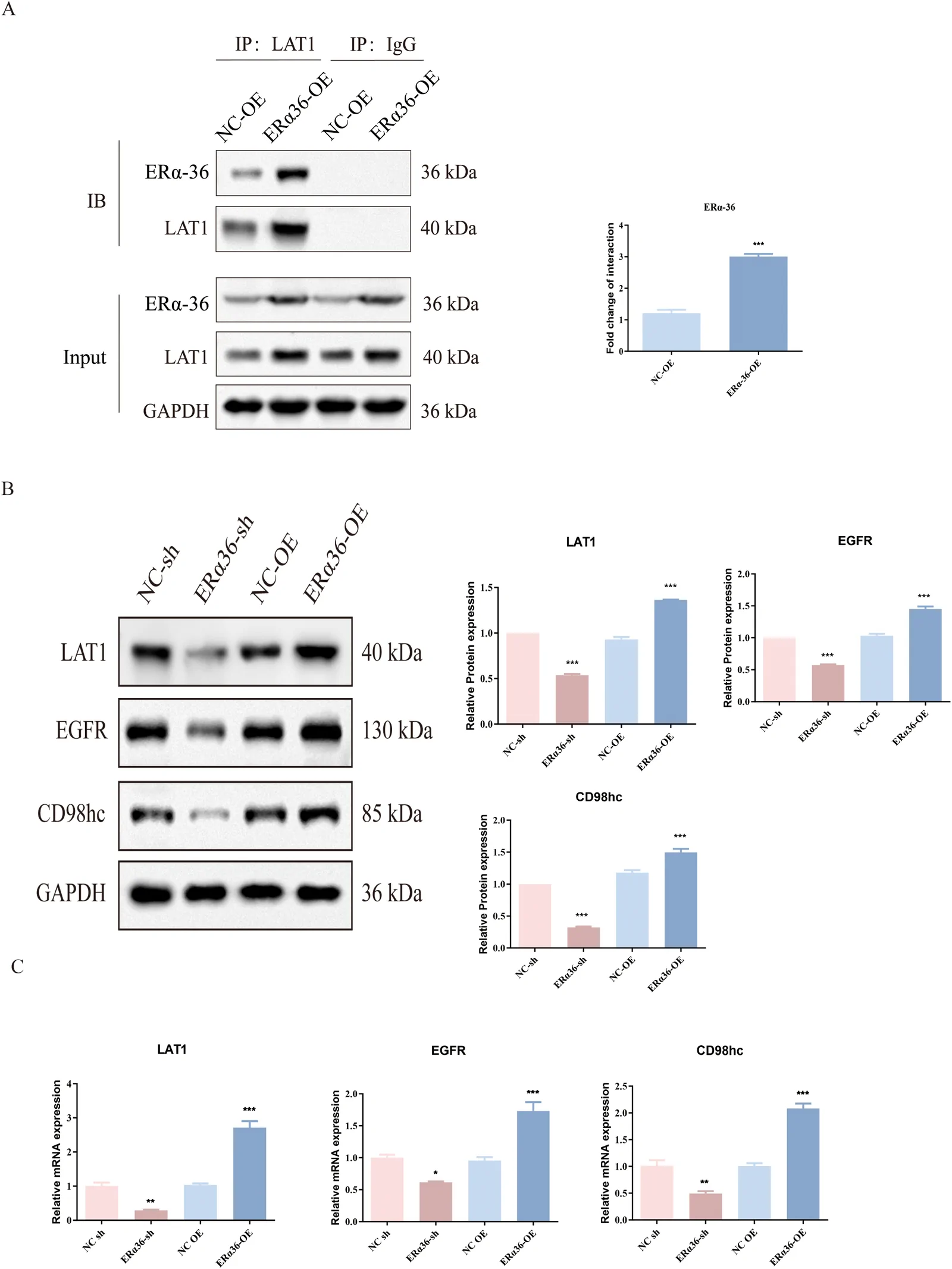
ERα36 physically interacts with LAT1 and is associated with CD98hc and EGFR expression. **(A)** Co-immunoprecipitation (Co-IP) experiments were conducted to verify the protein interaction between ERα36 and LAT1. In ERα36 overexpression (OE) and control cells, ERα36 antibody was used for immunoprecipitation, and Western blot was performed to detect the co-precipitated LAT1 protein. The Input group served as the corresponding total protein sample control. **(B)** Western blot was used to detect the protein expression levels of the key LAT1 cotransporter CD98hc (SLC3A2) and epidermal growth factor receptor (EGFR) under ERα36 knockdown (sh2) and overexpression (OE) conditions. GAPDH was used as the loading control. **(C)** qRT-PCR was employed to examine the effects of ERα36 expression alterations on the mRNA expression of CD98hc (SLC3A2) and EGFR. Data represent the mean ± standard deviation of three independent experiments, *P < 0.05, **P < 0.01, ***P < 0.001.

To investigate the molecular relationship between ERα36 and LAT1, (co-IP) was performed in SiHa cells. As shown in **Figure 4A**, LAT1 was specifically enriched in ERα36 immunoprecipitates from ERα36-overexpressing SiHa cells, whereas no detectable LAT1 signal was observed in control IgG pulldowns, suggesting a physical interaction between ERα36 and LAT1 in cervical cancer cells.

To assess whether ERα36 modulates downstream components of the LAT1 signaling axis, we examined CD98hc and EGFR expression upon ERα36 perturbation. Western blot analysis revealed that ERα36 overexpression significantly increased CD98hc and EGFR protein levels, while ERα36 knockdown markedly decreased both (**Figure 4B**). qRT-PCR confirmed that these changes were accompanied by parallel alterations in mRNA abundance: CD98hc and EGFR transcripts were upregulated upon ERα36 overexpression and downregulated upon knockdown (**Figure 4C**). Collectively, these findings demonstrate that ERα36 positively correlates with the expression of CD98hc and EGFR, two essential functional partners of LAT1,at both protein and mRNA levels. Thus, ERα36 engages LAT1 through both direct protein–protein interaction and coordinated transcriptional control of its coregulatory effectors, suggesting a role in modulating the LAT1-centered oncogenic network in cervical cancer.

### 3.5 ERα36 depletion attenuates BNCT-induced cytotoxicity and modulates cellular sensitivity to boron neutron capture therapy

**Figure 5:**
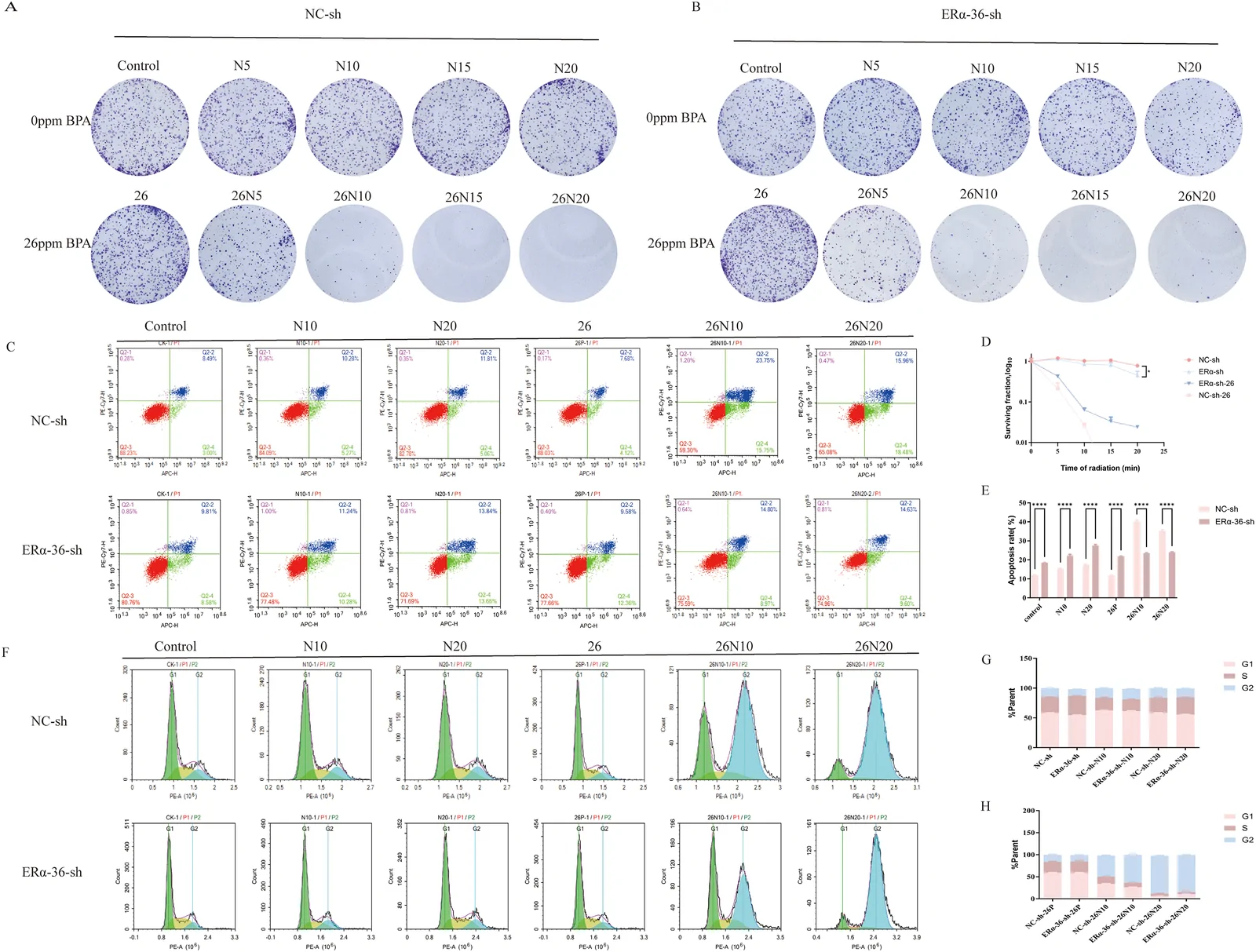
Knockdown of ERα36 reduces the sensitivity of cervical cancer cells to BNCT. **(A, B)** Representative images of the colony formation assay. The colony formation of NC-sh **(A)** and ERα36-sh **(B)** cells under different treatment conditions is shown: untreated control (N0), neutron irradiation alone for 10 min (N10) or 20 min (N20), BPA alone at 26 ppm (26), and BPA combined with neutron irradiation for 10 min (26N10) or 20 min (26N20). **(C)** Representative scatter plots of Annexin V-FITC/PI double staining flow cytometry for cell apoptosis. The apoptosis distribution of NC-sh and ERα36-sh cells under different BNCT treatment conditions is shown. **(D)** Quantitative analysis of the colony formation assay. Data are presented as the colony formation rate (% of control) and are the mean ± standard deviation of three independent experiments. Two-way ANOVA (genotype × treatment) with Tukey’s post-hoc test was applied; interaction P values are shown. *P < 0.05. **(E)** Quantitative analysis of apoptosis assays. Data are presented as the percentage of apoptotic cells (normalized to the corresponding untreated genotype control) and are the mean ± SD of three independent experiments. Two-way ANOVA (genotype × treatment) with Tukey’s post-hoc test was applied; interaction P values are shown. ****P < 0.0001. **(F, G)** Representative histograms of cell cycle analysis. The DNA content distribution of NC-sh **(F)** and ERα36-sh **(G)** cells under different treatment conditions is shown. **(H)** Quantitative analysis of cell cycle distribution. Data show the mean ± standard deviation of the proportion of cells in G1 phase, S phase, and G2/M phase under each treatment condition (n = 3).

Our previous studies have demonstrated the potential efficacy of boron neutron capture therapy (BNCT) in cervical cancer (24, 25).To further investigate the functional role of ERα36 in this therapeutic modality, we first examined whether ERα36 knockdown affects BPA uptake. Intracellular ¹^0^B concentration was measured using inductively coupled plasma-atomic emission spectroscopy (ICP-AES) in NC-sh and ERα36-sh SiHa cells after BPA incubation at three different concentrations (13 ppm, 26 ppm, and 39 ppm) for 6 hours. As shown in **Supplementary Figure 1**, ERα36 knockdown significantly reduced cellular ¹^0^B uptake at all three BPA concentrations compared with NC-sh controls (P < 0.05 for each concentration), indicating that ERα36 depletion impairs BPA uptake capacity, likely through downregulation of LAT1 expression. We then subjected ERα36-stably knocked-down SiHa cells (ERα36-sh) and non-targeting control cells (NC-sh) to a defined panel of treatment conditions: untreated control (N0); neutron irradiation alone (10 min: N10; 20 min: N20); BPA alone (26 ppm: BPA); and BPA combined with neutron irradiation (26 ppm + 10 min: BPA+N10; 26 ppm + 20 min: BPA+N20). Colony formation assays revealed that under neutron irradiation alone (N10, N20), ERα36 knockdown induced only a marginal reduction in clonogenic survival; in contrast, in the BPA–neutron combination groups (BPA+N10, BPA+N20), ERα36-sh cells exhibited significantly higher colony-forming capacity than NC-sh controls (**Figures 5A, B, D**), indicating that ERα36 depletion attenuates BNCT-induced cytotoxicity. Consistent with this, apoptosis analysis showed that although ERα36 knockdown alone increased basal apoptosis (**Figures 5C, E**), it markedly blunted BNCT-triggered apoptosis—specifically, the apoptosis rate in ERα36-sh cells was significantly lower than in NC-sh cells following BPA+N20 treatment (**Figures 5C, E**). Cell cycle profiling further uncovered context-dependent effects: under basal (N0) or neutron-only (N20) conditions, ERα36 knockdown induced S-phase accumulation—suggestive of replication stress or S-phase arrest—whereas under BNCT conditions (26N20), the S-phase fraction was significantly reduced in ERα36-sh versus NC-sh cells (**Figures 5F–H**), revealing a reversal of the S-phase response upon BNCT exposure. Collectively, these findings demonstrate that ERα36 expression level critically determines cellular sensitivity to BNCT in cervical cancer. ERα36 depletion impairs BPA uptake and attenuates BNCT efficacy by dampening apoptotic induction and altering cell cycle dynamics, suggesting that ERα36 may serve as a mechanistically relevant regulator of BNCT response.

### 3.6 Preliminary exploration of ERα36 and LAT1 as therapeutic targets for AB BNCT treatment

**Figure 6:**
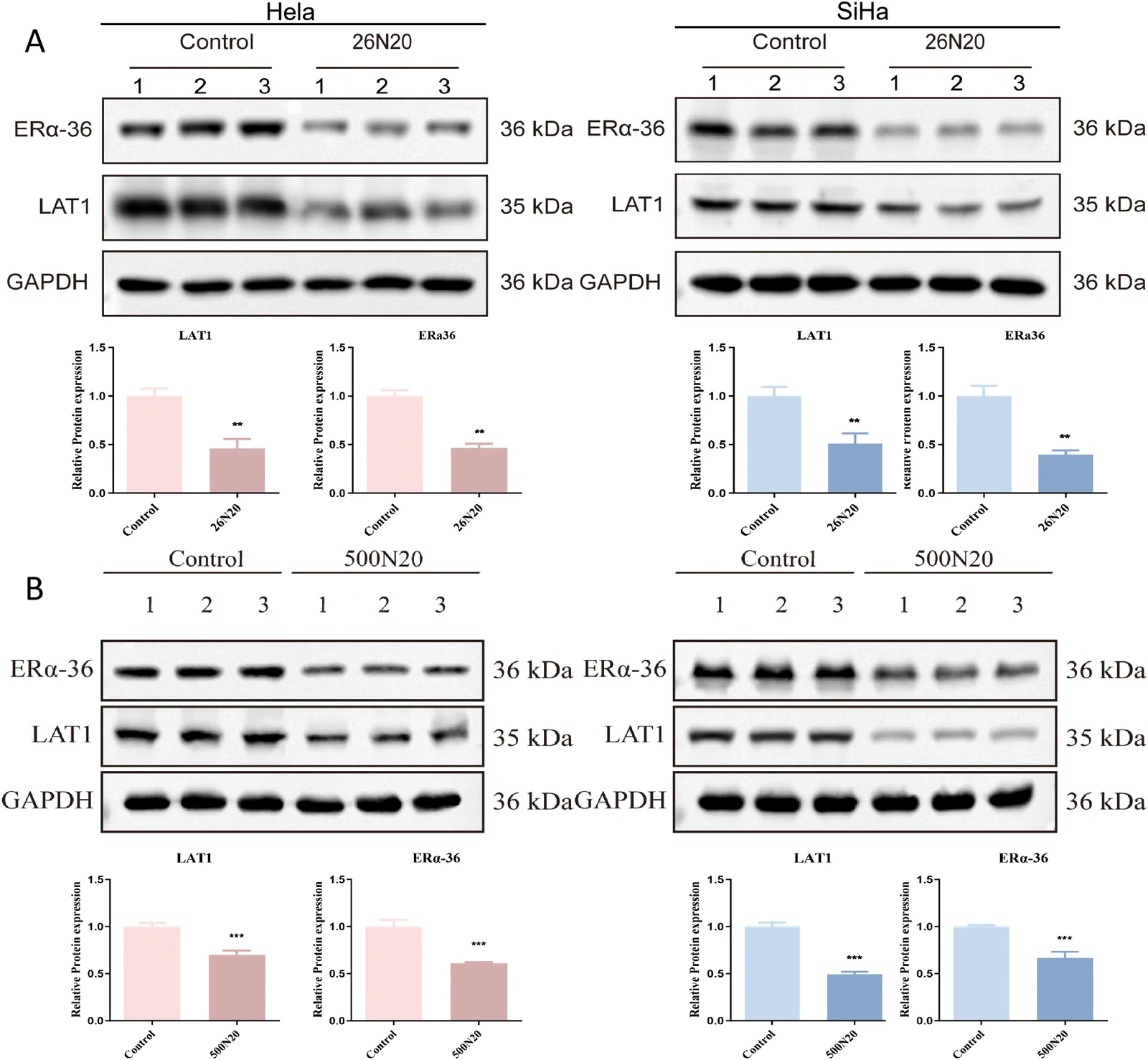
Western Blot analysis shows the changes in ERα36 and LAT1 protein expressions in cells and tumor tissues after BNCT treatment. **(A)** Comparison of ERα36 and LAT1 protein levels between the 26N20 cell group and the control group; **(B)** Comparison of ERα36 and LAT1 protein levels between the 500N20 tumor tissue group and the control group. Compared with the control group, **P<0.01, ***P<0.001.

To investigate the dynamic changes in the protein expression of ERα36 and LAT1 after AB BNCT treatment, this study used two cell lines, cervical adenocarcinoma cells HeLa and cervical squamous cell carcinoma SiHa. The cells were incubated with 26 ppm BPA for 6 hours and then subjected to AB BNCT irradiation. At 48 hours after irradiation, Western blot analysis showed that the protein levels of ERα36 and LAT1 in both cell lines significantly decreased (**Figure 6A**). This result was further verified in a subcutaneous tumor-bearing nude mouse model. The tumor-bearing mice were injected intravenously with 500 mg/kg BPA once, and then subjected to AB BNCT irradiation 1 hour later. Tumor tissues were collected 14 days after irradiation, and Western blot analysis confirmed that the protein expression of ERα36 and LAT1 was significantly reduced (**Figure 6B**). In summary, consistent downregulation of ERα36 and LAT1 co-expression was observed in both cell and animal models after AB BNCT treatment, suggesting that these two proteins can be candidate biomarkers for monitoring treatment response. In our previously published study (24), we demonstrated that BPA+neutron treatment significantly inhibited tumor growth compared with controls in the same xenograft model, confirming the *in vivo* efficacy of BNCT in this cervical cancer model. Consistent with the *in vitro* findings, *in vivo*, BNCT treatment was associated with decreased ERα36 and LAT1 protein expression in tumor tissues, suggesting that these two proteins may serve as candidate biomarkers for monitoring treatment response. However, we acknowledge that these data do not establish that ERα36 regulates BNCT sensitivity *in vivo*, and further loss-of-function or gain-of-function studies are needed to address this question.

## 4 Discussion

Cervical cancer remains a leading cause of cancer-related morbidity and mortality among women globally, and patients with advanced or metastatic disease continue to face a critical unmet need for effective targeted therapies. Integrating analyses of public omics databases, clinical tissue microarray validation, and comprehensive *in vitro* functional assays, this study reveals a correlation between ERα36, SLC7A5/LAT1 expression, and cervical cancer malignancy and BNCT sensitivity for the first time. Critically, this work suggests a potential link between estrogen non-genomic signaling and amino acid metabolic reprogramming, offering a novel conceptual framework that advances our understanding of cervical cancer biology and may inform molecular stratification, prognostic refinement, and BNCT response prediction.

SLC7A5/LAT1, a high-capacity transporter of essential large neutral amino acids such as leucine, phenylalanine, and tryptophan, is frequently overexpressed across diverse solid tumors and is tightly coupled to oncogenic growth signaling (26, 27). Consistent with this, our TCGA-based analysis confirmed significantly elevated SLC7A5 mRNA expression in cervical carcinoma tissues relative to normal cervical epithelium, and further revealed that high SLC7A5 expression strongly correlates with distant metastasis (M stage) and is associated with shorter overall survival. Yet, the upstream transcriptional or post-translational regulators responsible for LAT1 dysregulation in cervical cancer have remained elusive. Here, we demonstrate a robust positive correlation between ERα36 and SLC7A5 protein expression in clinical cervical cancer specimens, with clear spatial co-localization observed by multiplex immunofluorescence within the tumor microenvironment, thereby suggesting potential functional crosstalk and implicating ERα36 as a possible upstream correlate of LAT1. Importantly, multivariate Cox proportional hazards regression identified the ERα36+/SLC7A5+ dual-positive phenotype as an independent prognostic indicator, distinct from conventional clinicopathological variables such as age, T stage, and N stage, thereby highlighting the added value of spatially resolved, multi-marker phenotyping for precision risk assessment in cervical cancer.

ERα36, a membrane-localized splice variant of estrogen receptor α (ERα), has been established as an oncogenic driver in multiple malignancies such as breast, gastric, and lung cancers through its capacity to initiate rapid non-genomic signaling (28). However, its direct functional linkage to metabolic transport networks remains unreported. In this study, multiplex immunofluorescence staining revealed significant upregulation of ERα36 in cervical cancer tissues, with expression strongly correlated with the proliferation marker Ki67. Complementary *in vitro* functional assays demonstrated that ERα36 governs core malignant phenotypes such as proliferation, migration, resistance to apoptosis, and clonogenic survival, and that it physically interacts with LAT1 and is associated with elevated its essential co-transporters CD98hc (SLC3A2) and EGFR. While prior work has documented ERα36’s ability to rapidly activate membrane-proximal kinase cascades such as those involving EGFR, Src, and AKT upon ligand stimulation (29), direct regulation of membrane transporter function by ERα36 had not been described.

Here, we present biochemical and transcriptional evidence demonstrating that ERα36 establishes a self-reinforcing regulatory circuit: its physical interaction with LAT1 coordinately upregulates CD98hc and EGFR expression, thereby forming a positive feedback loop—ERα36–LAT1/CD98hc–EGFR. This finding bridges two previously disparate paradigms: estrogen-mediated non-genomic signaling and amino acid–driven metabolic reprogramming, thereby providing a unified mechanistic framework for the coordinated integration of hormonal cues and nutrient acquisition signals in tumor cells. Under conditions of diminished canonical nuclear ERα activity, ERα36 may assume dominance as the principal estrogen sensor, coupling rapid signal transduction to metabolic infrastructure to sustain malignant growth at both energetic and biosynthetic levels (10, 13, 15, 30). Notably, CD98hc occupies a critical nodal position at the interface of “adhesion and mechanotransduction—nutrient transport” (31, 32); the coordinated downregulation of CD98hc and EGFR upon ERα36 knockdown thus positions ERα36 as a central membrane-integrated hub coordinating growth factor signaling and metabolic input.

A second major translational insight from this work is the identification of the ERα36–SLC7A5/LAT1 axis as a key determinant of BNCT sensitivity. BNCT is a biologically targeted radiotherapy whose therapeutic index critically depends on selective intratumoral accumulation of boron carriers—most notably BPA—via LAT1-mediated transport. Given LAT1’s well-established role as the primary BPA transporter and validated predictor of BNCT response across tumor types, our discovery that ERα36 knockdown attenuates BNCT cytotoxicity—evidenced by enhanced clonogenic survival, reduced apoptosis, and diminished S-phase retention following BPA–neutron irradiation (26N20)—was initially counterintuitive. Yet this observation reveals a crucial conceptual refinement: BNCT efficacy is not dictated solely by boron delivery, but rather emerges from the dynamic interplay between boron accumulation and the cellular capacity to execute damage response programs.

BNCT induces densely ionizing, high-linear energy transfer (high-LET) radiation damage, which poses uniquely challenging repair demands compared with conventional low-LET radiotherapy. Cell fate after BNCT therefore hinges on the integrated functionality of DNA damage sensors, cell cycle checkpoints, and pro-survival signaling networks (33). ERα36 sustains these protective pathways, particularly through EGFR/ERK- and PI3K/AKT-mediated survival signaling, across diverse cancer types (18), thereby enabling post-damage recovery and clonal regeneration. Consequently, ERα36 depletion impairs this damage tolerance machinery, leading to paradoxically reduced BNCT sensitivity—not attributable to diminished boron accumulation, but rather to the failure of tumor cells to mount an effective survival response against the lethal damage induced by BNCT. Moreover, the ERα36-dependent shift in S-phase distribution further suggests modulation of cell cycle checkpoint engagement in response to high-LET damage. Thus, ERα36 governs BNCT response primarily through regulation of post-irradiation survival and checkpoint adaptation—not through modulation of BPA uptake per se. These findings establish ERα36 as a promising predictive biomarker for BNCT resistance and a rational combinatorial target: high ERα36 expression may identify patients likely to exhibit suboptimal BNCT response, and pharmacologic inhibition of ERα36 alongside BNCT could represent a novel strategy to overcome intrinsic or acquired resistance.

This study has several limitations that warrant acknowledgment. First, the precise molecular mechanism underlying ERα36-mediated regulation of SLC7A5/LAT1 expression remains incompletely defined. Although our data demonstrate that ERα36 positively regulates both LAT1 and CD98hc expression, the regulatory layer—whether transcriptional, translational, or post-translational (e.g., via modulation of protein stability)—has not been resolved. Moreover, it is unknown whether this regulation involves canonical estrogen response elements or is mediated by alternative transcription factors such as HIF-1α or MYC. Definitive mechanistic insight will require follow-up studies employing chromatin immunoprecipitation (ChIP), luciferase reporter assays with wild-type and mutant promoter constructs, and RNA polymerase II occupancy analysis. Second, although our data demonstrate that ERα36 positively correlates with CD98hc and EGFR mRNA levels, the present findings do not establish direct transcriptional regulation. We have therefore softened our interpretation throughout the manuscript, stating that ERα36 “positively correlates with CD98hc and EGFR mRNA levels” rather than “transcriptionally upregulates.” Definitive evidence of transcriptional regulation would require promoter-based assays such as luciferase reporter experiments or ChIP-qPCR. We have included this as a limitation and acknowledge that future studies are needed to determine whether ERα36 directly regulates CD98hc and EGFR transcription or acts through indirect mechanisms. Third, the functional consequences of the direct ERα36–LAT1 protein interaction remain to be fully elucidated. Specifically, it is unclear whether complex formation modulates LAT1’s subcellular trafficking, membrane residency, substrate binding affinity, or transport turnover rate. These questions can be addressed using complementary approaches—including surface plasmon resonance for binding kinetics, confocal microscopy coupled with fluorescence recovery after photobleaching for membrane dynamics, and radiolabeled leucine/phenylalanine uptake assays under controlled metabolic conditions. Fourth, although our co-immunoprecipitation data suggest a physical interaction between ERα36 and LAT1, the present findings do not distinguish whether this interaction is direct or mediated through adaptor proteins or other components of a larger protein complex. While our data provide evidence for their association in the cellular context, definitive determination of a direct versus indirect interaction would require additional approaches such as proximity ligation assay (PLA), immunofluorescence co-localization with super-resolution microscopy, or domain-mapping studies using purified recombinant proteins or truncation mutants. Future studies employing these complementary techniques will be necessary to fully elucidate the molecular nature of the ERα36–LAT1 interaction and to determine whether it involves direct binding or is bridged by adaptor molecules. Fifth, the current findings are primarily derived from *in vitro* cellular models and lack validation in physiologically relevant *in vivo* systems. Future work should therefore extend these observations to immunocompromised mouse xenograft models—including both cell-line-derived and patient-derived xenografts (CDX and PDX)—to rigorously assess the impact of the ERα36–SLC7A5/LAT1 axis on tumor growth, metastatic dissemination, and BNCT response in a tissue-contextualized microenvironment. Additionally, we acknowledge that the mechanistic and BNCT experiments were primarily performed using a single shRNA clone in one cell line. Although the sh2 clone was selected from three independent shRNA sequences and the observed phenotypes were consistent across multiple functional assays, we have not independently confirmed that ERα36 knockdown does not alter ERα66 expression. Future studies using independent shRNA sequences, rescue experiments, ERα66-specific confirmation, and additional cervical cancer cell models (e.g., C33A, CaSki) are essential to further validate and generalize our findings. While we have demonstrated that ERα36 knockdown reduces BPA uptake, we acknowledge that LAT1 rescue or inhibition experiments are needed to definitively establish whether the BNCT effect is LAT1-dependent, and we have prioritized this as a future direction.

## 5 Conclusions

In summary, this study provides the first systematic characterization of the association between ERα36, SLC7A5/LAT1 expression, cervical cancer malignancy, and BNCT sensitivity. We observed that ERα36 physically interacts with LAT1 and is associated with elevated CD98hc and EGFR expression. ERα36 and SLC7A5 exhibit co-expression and spatial phenotypic aggregation within the cervical cancer tumor microenvironment, and their dual positivity may represent a candidate prognostic biomarker. Functionally, ERα36 depletion attenuates BNCT-induced cytotoxicity, suggesting a role in modulating BNCT response. These findings integrate estrogen non-genomic signaling with amino acid metabolic reprogramming, and position the ERα36–SLC7A5/LAT1 association as a potential nexus linking oncogenic phenotype to therapeutic vulnerability. This work lays a foundation for further exploration of ERα36–SLC7A5/LAT1-guided molecular stratification, prognostic assessment, and BNCT response prediction in cervical cancer. Future studies should validate ERα36 expression as a potential patient selection criterion for BNCT and evaluate targeted approaches in combination with BNCT.

## Data Availability

The datasets presented in this study can be found in online repositories. The names of the repository/repositories and accession number(s) can be found in the article/**Supplementary Material**.

## References

[1] Ni H Huang C Ran Z Li S Kuang C Zhang Y . Targeting HPV for the prevention, diagnosis, and treatment of cervical cancer. J Mol Cell Biol. (2025) 16:mjae046. doi: 10.1093/jmcb/mjae046 39402008 PMC12080229

[2] Lin H Wu C-H Fu H-C Ou Y-C . Recent advances in cervical cancer treatment: Innovations from early-stage to advanced disease. Taiwan J Obstet Gynecol. (2025) 64:608–15. doi: 10.1016/j.tjog.2025.04.006 40602955

[3] Burmeister CA Khan SF Schäfer G Mbatani N Adams T Moodley J . Cervical cancer therapies: Current challenges and future perspectives. Tumour Virus Res. (2022) 13:200238. doi: 10.1016/j.tvr.2022.200238 35460940 PMC9062473

[4] Kanai Y . Amino acid transporter LAT1 (SLC7A5) as a molecular target for cancer diagnosis and therapeutics. Pharmacol Ther. (2021) 230:107964. doi: 10.1016/j.pharmthera.2021.107964 34390745

[5] Nicklin P Bergman P Zhang B Triantafellow E Wang H Nyfeler B . Bidirectional transport of amino acids regulates mTOR and autophagy. Cell. (2009) 136:521–34. doi: 10.1016/j.cell.2008.11.044 19203585 PMC3733119

[6] Wang Q Holst J . L-type amino acid transport and cancer: targeting the mTORC1 pathway to inhibit neoplasia. Am J Cancer Res. (2015) 5:1281–94. PMC4473310 26101697

[7] Salisbury TB Arthur S . The regulation and function of the L-Type Amino Acid Transporter 1 (LAT1) in cancer. Int J Mol Sci. (2018) 19:2373. doi: 10.3390/ijms19082373 30103560 PMC6121554

[8] Zhang C Xu J Xue S Ye J . Prognostic value of L-Type Amino Acid Transporter 1 (LAT1) in various cancers: A meta-analysis. Mol Diagn Ther. (2020) 24:523–36. doi: 10.1007/s40291-020-00470-x 32410110

[9] Uno K Kuwabara H Terado Y Kojima K Kawakami T Kamma H . Divergent expression of L-type amino acid transporter 1 during uterine cervical carcinogenesis. Hum Pathol. (2011) 42:1660–6. doi: 10.1016/j.humpath.2011.01.013 21530999

[10] Chung S-H Franceschi S Lambert PF . Estrogen and ERalpha: culprits in cervical cancer? Trends Endocrinol Metab. (2010) 21:504–11. doi: 10.1016/j.tem.2010.03.005 20456973 PMC2914219

[11] Chung S-H Wiedmeyer K Shai A Korach KS Lambert PF . Requirement for estrogen receptor alpha in a mouse model for human papillomavirus-associated cervical cancer. Cancer Res. (2008) 68:9928–34. doi: 10.1158/0008-5472.CAN-08-2051 19047174 PMC2596671

[12] Chung S-H Shin MK Korach KS Lambert PF . Requirement for stromal estrogen receptor alpha in cervical neoplasia. Horm Cancer. (2012) 4:50–9. doi: 10.1007/s12672-012-0125-7 23065599 PMC3541456

[13] Sun Q Liang Y Zhang T Wang K Yang X . ER-α36 mediates estrogen-stimulated MAPK/ERK activation and regulates migration, invasion, proliferation in cervical cancer cells. Biochem Biophys Res Commun. (2017) 487:625–32. doi: 10.1016/j.bbrc.2017.04.105 28435071

[14] Wang C Zhang T Wang K Zhang S Sun Q Yang X . ER-α36 promotes the Malignant progression of cervical cancer mediated by estrogen via HMGA2. Front Oncol. (2021) 11:712849. doi: 10.3389/fonc.2021.712849 34336701 PMC8317436

[15] Zhang X Zhang A Zhang X Hu S Bao Z Zhang Y . ERa-36 instead of ERa mediates the stimulatory effects of estrogen on the expression of viral oncogenes HPV E6/E7 and the Malignant phenotypes in cervical cancer cells. Virus Res. (2021) 306:198602. doi: 10.1016/j.virusres.2021.198602 34662680

[16] Pagano MT Ortona E Dupuis ML . A role for estrogen receptor alpha36 in cancer progression. Front Endocrinol (Lausanne). (2020) 11:506. doi: 10.3389/fendo.2020.00506 32849292 PMC7411082

[17] Thiebaut C Konan H-P Guerquin M-J Chesnel A Livera G Le Romancer M . The Role of ERα36 in development and tumor malignancy. Int J Mol Sci. (2020) 21:4116. doi: 10.3390/ijms21114116 32526980 PMC7312586

[18] Yin L Zhang X-T Bian X-W Guo Y-M Wang Z-Y . Disruption of the ER-α36-EGFR/HER2 positive regulatory loops restores tamoxifen sensitivity in tamoxifen resistance breast cancer cells. PloS One. (2014) 9:e107369. doi: 10.1371/journal.pone.0107369 25203051 PMC4159333

[19] Monti Hughes A Hu N . Optimizing Boron Neutron Capture Therapy (BNCT) to treat cancer: An updated review on the latest developments on boron compounds and strategies. Cancers (Bsl). (2023) 15:4091. doi: 10.3390/cancers15164091 37627119 PMC10452654

[20] Mechetin GV Zharkov DO . DNA damage response and repair in boron neutron capture therapy. Genes (Bsl). (2023) 14:127. doi: 10.3390/genes14010127 36672868 PMC9859301

[21] Wongthai P Hagiwara K Miyoshi Y Wiriyasermkul P Wei L Ohgaki R . Boronophenylalanine, a boron delivery agent for boron neutron capture therapy, is transported by ATB0,+, LAT1 and LAT2. Cancer Sci. (2015) 106:279–86. doi: 10.1111/cas.12602 25580517 PMC4376436

[22] Watanabe T Sanada Y Hattori Y Suzuki M . Correlation between the expression of LAT1 in cancer cells and the potential efficacy of boron neutron capture therapy. J Radiat Res. (2023) 64:91–8. doi: 10.1093/jrr/rrac077 36371738 PMC9855323

[23] Terada S Tsunetoh S Tanaka Y Tanaka T Kashiwagi H Takata T . Boron uptake of boronophenylalanine and the effect of boron neutron capture therapy in cervical cancer cells. Appl Radiat Isot. (2023) 197:110792. doi: 10.1016/j.apradiso.2023.110792 37062147

[24] Zhang L Quan R Huang H Wu M Wang W Zhang L . Boronophenylalanine-mediated boron neutron capture therapy confers selective killing of cervical cancer by exploiting DNA repair deficiency. Cancer Biother Radiopharm. (2026), 10849785261426657. doi: 10.1177/10849785261426657 41817167

[25] Braun K Wolber G Waldeck W Pipkorn R Jenne J Rastert R . The enhancement of neutron irradiation of HeLa-S cervix carcinoma cells by cell-nucleus-addressed deca-p-boronophenylalanine. Eur J Med Chem. (2003) 38:587–95. doi: 10.1016/s0223-5234(03)00088-6 12832130

[26] Cavalieri S Nuzzolese I Lenoci D Lucchetta M Ficorilli M Orlandi E . LAT1 expression in head and neck cancer: a prognostic biomarker with potential relevance for BNCT. Front Oncol. (2025) 15:1678011. doi: 10.3389/fonc.2025.1678011 41487563 PMC12757229

[27] Kikuoka Y Aihara T Higashino M Kurisu Y Haginomori S-I Nihei K . Expression rate of LAT1 in high-grade parotid gland carcinoma and potential of BNCT as a treatment option for recurrent parotid gland carcinoma. Appl Radiat Isot. (2025) 218:111657. doi: 10.1016/j.apradiso.2025.111657 39837206

[28] Mahboobifard F Dargahi L Jorjani M Ramezani Tehrani F Pourgholami MH . The role of ERα36 in cell type-specific functions of estrogen and cancer development. Pharmacol Res. (2020) 163:105307. doi: 10.1016/j.phrs.2020.105307 33246174

[29] Poyonov MMU Bui ATN Lee S-Y Lee G-H Jeong H-G . ERα36 promotes MDR1-mediated Adriamycin resistance via non-genomic signaling in triple-negative breast cancer. Int J Mol Sci. (2025) 26:7200. doi: 10.3390/ijms26157200 40806331 PMC12346883

[30] Bo T Kobayashi S Inanami O Fujii J Nakajima O Ito T . LAT1 inhibitor JPH203 sensitizes cancer cells to radiation by enhancing radiation-induced cellular senescence. Transl Oncol. (2021) 14:101212. doi: 10.1016/j.tranon.2021.101212 34461558 PMC8405945

[31] Estrach S Lee S-A Boulter E Pisano S Errante A Tissot FS . CD98hc (SLC3A2) loss protects against ras-driven tumorigenesis by modulating integrin-mediated mechanotransduction. Cancer Res. (2014) 74:6878–89. doi: 10.1158/0008-5472.CAN-14-0579 25267066 PMC5120678

[32] Park E Kim H Yoon S Jang B . The role of CD98 heavy chain in cancer development. Histol Histopathol. (2024) 39:1557–64. doi: 10.14670/HH-18-749 38695393

[33] Wang W Zhang E Shan J Zhang M Cai R Li R . State-of-the-art boron clusters for boron neutron-capture therapy. Theranostics. (2026) 16:417–64. doi: 10.7150/thno.123376 41328345 PMC12665126

